# N-Degron-Based PROTAC Targeting PLK1: A Potential Therapeutic Strategy for Cervical Cancer

**DOI:** 10.3390/pharmaceutics17081027

**Published:** 2025-08-07

**Authors:** Pethaiah Gunasekaran, Sang Chul Shin, Yeon Sil Hwang, Jihyeon Lee, Yeo Kyung La, Min Su Yim, Hak Nam Kim, Tae Wan Kim, Eunjung Yang, Soo Jae Lee, Jung Min Yoon, Eunice EunKyeong Kim, Seob Jeon, Eun Kyoung Ryu, Jeong Kyu Bang

**Affiliations:** 1 Division of Magnetic Resonance, Korea Basic Science Institute (KBSI), Ochang, Cheongju 28119, Republic of Korea; gunaharaks@gmail.com (P.G.);; 2 Dandicure Inc., Ochang, Cheongju 28119, Republic of Korea; yshwang2@dandicure.com (Y.S.H.); jhlee4@dandicure.com (J.L.);; 3 Convergent Research Support Division, Technological Convergence Center, Korea Institute of Science and Technology (KIST), Seoul 02792, Republic of Korea; scshin84@kist.re.kr; 4 College of Pharmacy, Chungbuk National University, Cheongju 28160, Republic of Korea; 5 Korea Disease Control and Prevention Agency, National Institute of Health Center for Emerging Virus Research, Division of Emerging Virus and Vector Research, Cheongju 28159, Republic of Korea; 6 Future Innovation Medical Research Center, Soonchunhyang University Cheonan Hospital, Cheonan 31151, Republic of Korea; 7 Department of Obstetrics and Gynecology, College of Medicine, Soonchunhyang University Cheonan Hospital, Cheonan 31151, Republic of Koreasjeon4595@gmail.com (S.J.); 8 Biomedical Research Institute, Korea Institute of Science and Technology, Seoul 02792, Republic of Koreaeunice@kist.re.kr (E.E.K.)

**Keywords:** PLK1, PROTAC, N-end rule pathway, anticancer, N-degron

## Abstract

**Background**: Cervical cancer remains a major global health concern, with existing chemotherapy facing limited effectiveness owing to resistance. Polo-like kinase 1 (PLK1) overexpression in cervical cancer cells is a promising target for developing novel therapies to overcome chemoresistance and improve treatment efficacy. **Methods**: In this study, we developed a novel PROTAC, NC1, targeting PLK1 PBD via the N-end rule pathway. **Results**: This PROTAC effectively depleted the PLK1 protein in HeLa cells by inducing protein degradation. The crystal structure of the PBD-NC1 complex identified key PLK1 PBD binding interactions and isothermal titration calorimetry (ITC) confirmed a binding affinity of 6.06 µM between NC1 and PLK1 PBD. NC1 significantly decreased cell viability with an IC_50_ of 5.23 µM, induced G2/M phase arrest, and triggered apoptosis in HeLa cells. In vivo, NC1 suppressed tumor growth in a HeLa xenograft mouse model. **Conclusions**: This research highlights the potential of N-degron-based PROTACs targeting the PLK1 protein in cancer therapies, highlighting their potential in future cervical anticancer treatment strategies.

## 1. Introduction

According to the WHO, cervical cancer was the fourth most common cancer among women globally in 2022, with an estimated 660,000 new cases. Cervical cancer remains a considerable health threat, with 94% of the 350,000 deaths occurring in low- and middle-income countries [1]. Chemotherapy, particularly cisplatin, is the primary treatment for advanced or recurrent cervical carcinoma [2]. However, the response rate is limited to approximately 23% owing to chemoresistance. In addition, carboplatin, another platinum-based chemotherapy, is often used as an alternative for patients intolerant to cisplatin. However, chemotherapy can cause several side effects, such as nausea, fatigue, hair loss, and increased infection risk. Therefore, new strategies are needed to overcome this resistance and enhance both treatment outcomes and patient prognosis. Polo-like kinase 1 (PLK1) is overexpressed in cervical cancer cells, linking it to disease progression [3,4].

The Polo-like kinases (PLKs 1-5) are a family of serine/threonine protein kinases essential for cell cycle regulation and cell proliferation [5]. Among them, PLK1 is an attractive anticancer drug target owing to its overexpression in various human cancers. In humans, PLK1 regulates key processes during cell division, including mitotic entry, spindle formation, chromosome segregation, and cytokinesis [6]. Targeting PLK1 to reverse its oncogenic role in cancer cells is a promising anticancer strategy for inducing apoptosis [7]. PLK1 inhibitors target either the N-terminal catalytic kinase domain (KD) with ATP-binding sites or the C-terminal polo-box domain (PBD), which mediates interactions with phosphorylated substrates. Several KD-targeting inhibitors, such as BI2536, volasertib (BI6727), ON 01910, NMS-P937, and TAK-960, have been developed for various cancers and progressed to clinical trials [8]. In particular, volasertib notably suppresses tumor growth in human cervical cancer by inducing cell cycle arrest at the G2/M phase and apoptosis, accompanied by a decrease in PLK1 protein expression [9].

Although KD inhibitors demonstrate promising tumor suppression, their efficacy is limited by toxicity owing to cross-reactivity with other kinases that shared high sequence similarity. Alternatively targeting the PLK1 PBD offers greater specificity [10]. For example, peptide-based PLK1 PBD inhibitors, such as PLHSpT, and its derivatives exhibit strong potency [11]. However, their clinical potential is hindered by poor cell permeability and proteolytic instability. Although chemical modifications, including pegylation, alkyl capping, peptoid introduction, and cyclization, improve cell permeability, they increase production costs and often result in reduced potency [12].

**Figure 1:**
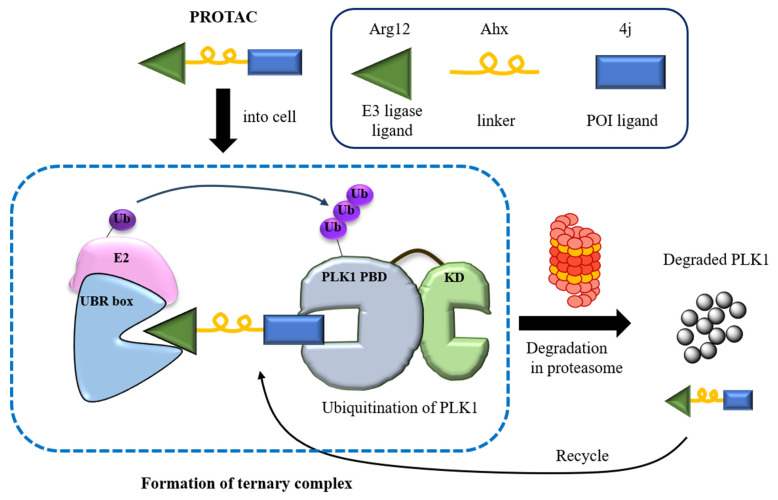
Illustration of the PROTAC, NC-1.

To address these issues, we considered Proteolysis Targeting Chimeras (PROTACs) as a promising tool to degrade PLK1 in cancer cells. We designed and developed NC1, a PROTAC combining PLK1-targeting inhibitor 4j [11] and UBR-box-targeting ligand Arg12, acting as an N-end rule pathway or N-degron inhibitor (Figure 1). PROTACs represent a new drug discovery paradigm by inducing target protein degradation via the ubiquitin-proteasome system. Unlike traditional drugs that act by occupying active sites and often require high doses, potentially causing side effects and resistance, PROTACs act via ubiquitin-mediated protein degradation [13]. Consequently, over 40 different targets PROTACs have been successfully degraded using PROTACS, addressing the limitations of traditional drug discovery methods [14]. PROTACs are designed with heterobifunctional ligands composed of a protein of interest (POI) ligand and a ubiquitin-ligase (E3 ligase) ligand, connected by a linker. PROTACs effectively degrade targets by forming a ternary complex that brings the target protein and an E3 ligase into proximity, enabling selective polyubiquitination and subsequent 26S proteasome degradation. Thus, PROTACs are recyclable, allowing continuous degradation of the target protein [15].

Although PROTACs have been successfully applied to different targets, several challenges need to be addressed to fully realize their potential in drug discovery. Despite over 800 E3 ubiquitin ligases exist in mammalian genomes [16], a small set, such as VHL, cereblon, Mdm2, and IAP, are commonly used in most protein degradations [17]. These E3 ligases have limited or low expression in the kidney, lung, and brain, complicating PROTAC development targeting cancers in those organs [18,19]. In addition, cereblon-based PROTACs can cause off-target effects, including degrading unintended proteins, such as IKZF1, IKZF3, and SALL4, raising concerns about their safety [20,21].

To address the current limitations, targeting the N-end rule pathway (N-degron pathway) is a promising PROTAC development strategy. This pathway, the first identified ubiquitin-mediated proteolysis system, regulates protein degradation to maintain proteostasis by eliminating abnormal proteins. The N-end rule correlates protein stability with N-terminal amino acids known as N-degrons. In the N-end rule pathway, N-degrons consist of destabilizing residues or degradation signals recognized by UBR protein components known as N-recognins, which are a class of E3-ligases [22]. Once recognized, N-degrons initiate polyubiquitination and subsequent degradation by the 26S proteasome. N-degrons are classified into Type 1 (basic amino acids such as Arg, Lys, His) and Type 2 (hydrophobic amino acids such as Phe, Leu, Trp, Tyr, Ile) [23]. The UBR box, a 70–80 residue domain, is found in seven mammalian UBR proteins (UBR1–7). Among these, UBR1 and UBR2 are highly homologous in sequence and size (approximately 200 kDa) and contain a conserved domain with UBR boxes that include Type 1 and Type 2 binding sites [24]. UBR1 was the first E3 ligase identified to interact with destabilizing residues [25].

The N-end rule pathway includes the Ac/N-end rule (acetylated N-terminal residues) and Arg/N-end rule (N-terminal Arg residues) pathways. The Arg/N-degron pathway regulates apoptosis, neurodegeneration, vascular development, and cell motility [23]. As UBR proteins are widely prevalent in most cells, selective degradation of a protein of interest can be strategically achieved by constructing a PROTACs molecule containing Arg/N- degron and a ligand for the protein of interest [26,27].

As part of our research on the discovery of N-degron-mediated PROTACs for PLK1 degradation, we present the first synthesis of NC1, a PROTAC targeting PLK1 via the N-degron pathway [19]. Structure–activity relationship (SAR) studies identified a potential Arg/N-degron recognized by UBR1 that induces PLK1 degradation. NC1 was evaluated in vitro for effects on cell viability, cell penetration, G2/M phase cell cycle arrest, and apoptosis in HeLa cells. In addition, vivo studies using a HeLa xenograft cervical cancer mouse model were conducted to further assess the therapeutic potential of NCI.

## 2. Materials and Methods

### 2.1. Chemistry

#### 2.1.1. General Methods

All chemicals and reagents used in this study were obtained from commercial suppliers, including Merck (Darmstadt, Germany), TCI (Tokyo, Japan), and Across Organic (Waltham, MA, USA), and were utilized without further purification. Amino acids were sourced from Bed-tech Korea (Seoul, Republic of Korea). Organic solvents with a purity exceeding 99.9% were procured from Aldrich (Darmstadt, Germany) and employed as received. Thin-layer chromatography (TLC) was carried out using Merck silica gel 60 F254 coated aluminum plates (Darmstadt, Germany), and detection was performed under UV light followed by staining with phosphomolybdic Acid (PMA), potassium permanganate or ninhydrin. All compounds were purified using column chromatography was performed using Merck silica gel 60 (70–230 mesh or 230–400 mesh). Bruker DRX-400 (Bruker, Ettlingen, Baden-Württemberg, Germany) and DRX-500 instruments (Bruker, Ettlingen, Baden-Württemberg, Germany) were used to record the NMR analyses, including both ^1^H and ^13^C spectra. Chemical shifts (δ) are given in parts per million (ppm), referenced to an internal standard, and coupling constants (J) are provided in hertz (Hz). Shimadzu (MALDI-TOF) mass spectrometer (Shimadzu, Kyoto, Japan) was used for determining the Peptide mass.

#### 2.1.2. Peptide Synthesis

All peptides were synthesized via solid-phase peptide synthesis (SPPS) on Rink amide resin (100 mg, initial loading 0.61 mmol/g) using Fmoc amino acids [12]. The resin was pre-swelled in N,N-dimethylformamide (DMF) for 45 min before deprotecting the Fmoc group with 20% piperidine in DMF (1 × 10 min, 2 × 3 min). The sequence extension was carried out by treating the resin with amino acid (5.0 equivalent), 2-(1H-benzotriazole-1-yl)-1,1,3,3-tetramethyluronium hexafluorophosphate (HBTU) (5.0 equivalent), 1-hydroxybenzotriazole (HOBt) (5.0 equivalent), and diisopropylethyl amine (DIEA) (10.0 equivalent) in DMF (2 mL) and agitated for 2 h in a vortex stirrer. After coupling, the resin was washed with DMF and Fmoc deprotection was repeated using 20% piperidine in DMF (1 × 10 min, 2 × 3 min) to couple with the next amino acid. This cycle was continued until completion of the peptide sequence. At the end of the final amino acid sequence, the final Fmoc group was removed, and the resin was washed with DMF, methanol, dichloromethane and ether. The resin was finally dried under vacuum. Peptide cleavage from the resin was achieved by treating with trifluoroacetic acid (TFA, 2 mL per 100 mg of resin) containing 5% triisopropylsilane (TIS) and 5% water for 2 h. The resulting mixture was poured into cold diethyl ether, resulted in the formation of precipitate, which is centrifuged and isolated. The crude product was purified by reverse-phase HPLC using mobile phase water/acetonitrile with 0.05% TFA and Vydac C18 column (20 mm × 250 mm, 15 µm,) detection at 230–280 nm. The collected fractions were lyophilized. Analytical purity (>98%) was confirmed via RP-HPLC using an analytical Vydac C18 column (4.6 mm × 250 mm, 300 Å, 5 µm particle size and a linear gradient of 0.05% TFA in water and acetonitrile (10–90% over 30 min, 1.5 mL/min, 25 °C, 280 nm detection)). The synthesized peptides were characterized using MALDI-TOF MS.

### 2.2. MALDI-TOF Mass Spectrometry Analysis of Synthesized Peptides

1. NC1: calculated mass for C_116_H_216_N_57_O_23_P, *m/z* 2806.72; observed *m/z* 2807.82 [M + H]+.
2. Arg1-K(biotin): calculated mass for C_22_H_41_N_9_O_4_S, *m/z* 527.30; observed *m/z* 528.38 [M + H]+.
3. Arg4-K(biotin): calculated mass for C_40_H_77_N_21_O_7_S, *m/z* 995.60; observed *m/z* 996.71 [M + H]+.
4. Arg8-K(biotin): calculated mass for C_64_H_125_N_37_O_11_S, *m/z* 1620.01; observed *m/z* 1621.22 [M + H]+.
5. Arg10-K(biotin): calculated mass for C_76_H_149_N_45_O_13_S, *m/z* 932.21; observed *m/z* 1933.522 [M + H]+.
6. Arg12-K(biotin): calculated mass for C_88_H_173_N_53_O_15_S, *m/z* 2244.41; observed *m/z* 2245.569 [M + H]+.
7. Ala4Arg8-ahx-4j: calculated mass for C_104_H_188_N_45_O_23_S, *m/z* 2467.87; observed *m/z* 2468.97 [M + H]+.

### 2.3. Pull-Down Assay

UBR constructs were obtained through PCR amplification and subsequently inserted into the pcDNA3.1/His_6_ expression vector [28]. HEK293T cells were transiently transfected with these plasmids using Lipofectamine 2000 (Invitrogen, Waltham, MA, USA) according to the manufacturer’s instructions. After 24 h, cells were collected, rinsed twice with ice-cold phosphate-buffered saline (PBS), and lysed using a hypotonic lysis buffer consisting of 10 mM KCl, 15 mM MgCl_2_, and 10 mM HEPES (pH 7.9), supplemented freshly with protease and phosphatase inhibitors (0.01 g/mL). The protein concentration in the lysates was quantified using a BCA assay.

Biotinylated peptides (R1, R4, R8, R10, R12, and NC1) were conjugated with streptavidin agarose beads. An amount of 300 µg of total cell lysate protein was diluted in a binding buffer containing 0.05% Tween-20, 10% glycerol, 0.2 M KCl, and 20 mM HEPES (pH 7.9), and incubated with peptide-bound beads at 4 °C for 3 h under gentle rotation. Following incubation, the beads were washed five times with cold binding buffer to remove unbound material. Proteins retained on the resin were eluted by boiling the beads in SDS sample buffer at 100 °C for 5 min. The presence of UBR proteins associated with the NC1 peptide was confirmed via immunoblotting as described previously [29].

### 2.4. Protein Expression and Purification

The mouse PLK1 in Polo box domain (PBD) (Swiss Prot entry Q07832, residues 367–603) was expressed as a recombinant protein containing an N-terminal 6XHis tag in the pET28a vector and a tobacco etch virus (TEV) protease cleavage was engineered between the affinity tag and PLK1 PBD. PLK1 PBD protein expression was induced by adding 0.1 mM IPTG and incubating the cells overnight at 18 °C. Following induction, the cells were collected by centrifugation and lysed via sonication after being resuspended in buffer composed of 20 mM HEPES (pH 7.5), 200 mM NaCl, 2 mM β-mercaptoethanol, and 1 mM PMSF. The lysate was clarified by centrifugation at 18,000 rpm for 40 min at 4 °C. The resulting supernatant was subjected to immobilized metal affinity chromatography (IMAC) using a Ni^2+^-NTA HiTrap column (GE Healthcare), and the protein was eluted using an imidazole gradient ranging from 25 to 500 mM. For further purification, size-exclusion chromatography was conducted on a HiLoad 26/60 Superdex-75 column equilibrated with 20 mM HEPES (pH 7.2), 150 mM NaCl, and 2 mM DTT. PLK1 PBD protein was purified the purity of the proteins at each stage was checked on 15% SDS-PAGE. The purified PLK1 PBD protein was concentrated to 10 mg/mL using a VIVASPIN20 (Sartorius, Göttingen, Germany) concentrator and stored at −80 °C until use.

### 2.5. Crystallization, Data Collection, Structure Solution, and Refinement

Crystallization trials for the PLK1 PBD-NC1 complex were conducted using the sitting-drop vapor-diffusion method at 22 °C with a Mosquito robotic crystallization system (TTP Labtech, San Diego, CA, USA). Prior to crystallization, the purified PLK1 PBD protein was concentrated to ~5 mg/mL, and the NC1 compound was mixed at a molar ratio of 1:5 and incubated overnight at 4 °C. Crystals were obtained by mixing equal volumes of protein–ligand complex with reservoir buffer containing 0.1 M sodium malonate (pH 5.4), 22% PEG3350, and 200 mM NaCl in 20 mM HEPES (pH 7.5). Cryoprotection was achieved by supplementing the reservoir buffer with 30% ethylene glycol prior to flash freezing in liquid nitrogen. X-ray diffraction data were collected at 100 K at beamline 5C (Pohang Light Source, Pohang, Gyeongbuk, Republic of Korea) using an ADSC Quantum 315r detector. Crystals belonged to space group P2_1_ with unit-cell dimensions a = 35.67, b = 51.45, c = 57.41 Å, α = γ = 90°, β = 100.89°. The crystal diffracted X-ray to 1.95 Å Bragg spacings, and the completeness was less than 97.6%. Data were indexed and scaled using the HKL2000 suite (1). Molecular replacement was carried out with PHENIX (2) using the coordinates of 3H1K as a template. Model building and refinement were performed with COOT (3) and PHENIX (2), respectively. The final structure was validated using PROCHECK (4), and structural figures were rendered in PyMOL. (5). The statistics of data collection and refinement are summarized in Table S1.

### 2.6. Isothermal Titration Calorimetry (ITC)

ITC measurements were carried out using a MicroCal ITC200 calorimeter (GE Healthcare, Chicago, IL, USA) at 25 °C. Prior to the assay, protein solutions were centrifuged at 18,000× *g* for 5 min at 4 °C to remove particulates. PLK1 PBD was placed in the sample cell at a concentration of 20 µM, while the ligand (NC1) solution at 200 µM was loaded into the injection syringe. Protein samples were dialyzed overnight against a buffer containing 20 mM HEPES (pH 7.5), 200 mM NaCl, and 5% DMSO. A series of 2 µL ligand injections were performed with 150 s intervals between each. Thermodynamic binding parameters, including the binding constant (Kd), stoichiometry (n), and enthalpy change (ΔH), were extracted by fitting the data to a one-site interaction model using ORIGIN 7.0 software with nonlinear regression analysis. Baseline corrections were applied using the corresponding buffer titration.

### 2.7. Compound Treatment and Immunoblotting

HeLa cells were seeded in 12-well plates with 1 × 10^5^ cells per well and allowed to stay for overnight. Various concentrations (0, 1, 2, 3, 4, and 5 µM) of NC1 were added to HeLa cells in 12-well plates and incubated for 24 h. An amount of 5 µM of MG132 was added to the cells and incubated for 8 h to evaluate the proteasome inhibition. before harvesting. Following treatment, cells were lysed and total proteins were extracted for immunoblot analysis of PLK1 expression [30]. Protein samples were separated by SDS-PAGE and transferred onto PVDF membranes. PLK1 was detected using a mouse-derived monoclonal antibody, with β-actin used as an internal control to ensure equal loading [31].

### 2.8. In Vivo Antitumor Activity

The antitumor efficacy of NC1 was assessed in a HeLa xenograft mouse model, as reported [10]. Briefly, 5 × 10^6^ HeLa cells were subcutaneously injected into the right thigh region of immunodeficient nude mice. When tumors reached an approximate volume of 100 mm^3^ (calculated using the formula: volume = 0.52 × length × width^2^) [32], mice were randomly assigned to treatment groups. NC1 (4 mg/kg) or PBS (vehicle control) was administered via tail vein injection at intervals of 3–4 days over a period of 29 days. Tumor volumes were measured regularly using calipers. All animal procedures were approved by the KBSI Institutional Animal Care and Use Committee (Protocol No. KBSI-IACUC-24-15) and were conducted in accordance with institutional and governmental regulations.

### 2.9. Cell Viability Assay

HeLa cells were cultured in Dulbecco’s modified Eagle’s medium (DMEM) supplemented with 10% fetal bovine serum and maintained at 37 °C in a 5% CO_2_ atmosphere. HeLa cells were seeded into 96-well plates at a density of 5 × 10^3^ cells per well in 100 µL of medium. Once they reached ~90% confluence, they were treated with different concentrations (0.3–12 µM) of Arg12, 4j, A4R8-4j, and NC1 for 24 h. Cell viability was assessed using the Enhanced MTT Cell Viability Assay Kit (DoGenBio, Seoul, Republic of Korea), following the manufacturer’s instructions. After a 2 h incubation with 10 µL of MTT solution, absorbance at 450 nm was analyzed using a microplate reader (Molecular Devices, San Jose, CA, USA) [33].

### 2.10. Morphological Assessment of NC1 Treated Cancer Cells

To assess the morphological changes induced by the compounds, HeLa cells were seeded in 24-well plates (4 × 10^4^ cells per well) and cultured for 30 h at 37 °C. After switching to serum-free medium, cells were treated with 6 µM of Arg12, 4j, A4R8-4j, or NC1. After 24 h of incubation, cellular morphology was visualized using phase contrast microscopy (Nikon ECLIPSE TS100, Tokyo, Japan) [33].

### 2.11. Cellular Uptake of FITC-Conjugated Compounds

For fluorescence-based internalization studies, HeLa cells were grown on plastic coverslips (Nunc™ Thermanox™, Thermo Fisher Scientific, Waltham, MA, USA) in 24-well plates at a density of 4 × 10^4^ cells/well for 24 h. Cells were treated with 6 µM of FITC-conjugated A4R8-4j or NC1 for 24 h. After treatment, coverslips were washed three times with PBS and counterstained with propidium iodide (PI, 1 µg/mL) for 10 min. Cells were then mounted and imaged using a confocal laser scanning microscope (LSM710, Carl Zeiss, Germany), and images were analyzed using ZEN2009 (version 5.5 SP1) software [10].

### 2.12. Analysis of G2/M Phase Cell Cycle Arrest by Flow Cytometry

HeLa cells (3.5 × 10^5^ cells/well) were seeded into 6-well plates and grown to 80–90% confluence. Cells were treated with 6 µM of each compound (Arg12, 4j, A4R8-4j, and NC1) for 24 h. Both medium and floating cells were collected, washed with PBS three times, and fixed in 70% cold ethanol. Further, cells were washed again with PBS and stained with a solution containing PI (1 µg/mL) and RNase A (1 µg/mL) at 37 °C for 30 min. The stained cells were analyzed using a CytoFLEX flow cytometer (Beckman Coulter, Brea, CA, USA), and data were processed with FlowJo v10.10 [10].

### 2.13. Evaluation of Cancer Cell Apoptosis Using Fluorescence Apoptosis Assay

HeLa cells were seeded into 96-well plates at 5 × 10^3^ cells/well and allowed to reach approximately 95% confluence. Cells were then treated with 6 µM of Arg12, 4j, A4R8-4j, or NC1 for 24 h. Following treatment, a staining solution was prepared by mixing 3 µL of the LIVE/DEAD™ Viability/Cytotoxicity Kit (Invitrogen, Waltham, MA, USA) reagents in 1 mL of PBS. A 100 µL aliquot of this solution was added to each well. After 30 min of incubation at room temperature, fluorescent images were captured using a Nikon ECLIPSE TS100 fluorescence microscope (Nikon, Tokyo, Japan) [33].

### 2.14. Apoptosis Detection by Flow Cytometry

HeLa cells were plated in 24-well plates (4 × 10^4^ cells/well) and allowed to culture for overnight. Cells were treated with 6 µM of Arg12, 4j, A4R8-4j, and NC1. After incubation for 24 h, both medium and adherent cells were collected by trypsinization, washed thoroughly with PBS (five times), and resuspended in binding buffer (10 mM HEPES, 100 mM NaCl, 2.5 mM CaCl_2_). Suspended cells were stained with Annexin V APC (5 µg/mL) and PI (1 µg/mL) for 30 min at room temperature in the dark. Samples were analyzed using flow cytometry (CytoFLEX, Beckman Coulter), and the proportion of apoptotic cells was quantified with FlowJo software [33].

### 2.15. Statistical Analysis

Data are presented as the mean ± SEM and analyzed DC_50_ (concentration that induced half-maximal degradation of PLK1) and IC_50_ (the concentration that inhibits cell growth by 50%) using GraphPad Prism 5.0 The statistical test was performed through “*t*-test (** *p* < 0.005, *** *p* < 0.001, **** *p* < 0.0005)”.

## 3. Results

### 3.1. Design and Synthesis of PROTAC, NC1

**Figure 2:**
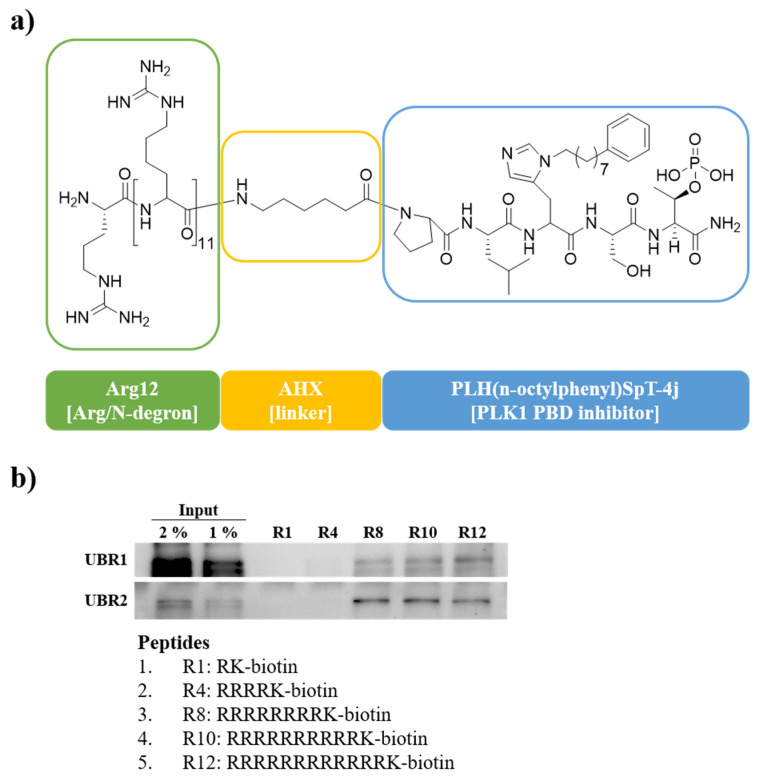
(**a**) Design strategy of the PROTAC, NC1. Structure of NC1 featuring Arg12 and PLH(*n*-octylphenyl)SpT (4j) [11] as warheads targeting N-degron and PLK1, respectively. Aminohexanoic acid (AHX) was used as the linker. (**b**) A biotinylated arginine pull-down assay was performed using biotinylated arginine sequences. The pull down assay was conducted on lysates from HEK293T cells overexpressing UBR1 or UBR2 using Rn.

The PROTAC drug is designed to target PLK1 PBD as the POI, while recruiting the UBR box as the E3 ligase. It consists of a linker that connects the POI and the E3 ligase. The 4j peptide serves as the ligand for PLK1 PBD, whereas Arg12 binds to the UBR box. These two components are linked by an aminohexanoic acid (Ahx) linker, as illustrated in Figure 2a. Among the PLHSpT derivatives, PLH(*n*-octylphenyl)SpT (4j) is the most potent PLK1 PBD inhibitor [11]. Incorporating an arginine residue at the N-terminal of PROTAC is an effective strategy for developing new N-degron pathways, as arginine is a potential type 1 degron recognized by N-recognins [19]. Moreover, the expanding application of cationic arginine-rich peptides as cell-penetrating peptides (CPPs), owing to their reduced toxicity and exceptional ability to cross cancer cell membranes, prompted the incorporation of multiple arginine residues (Arg12) as cell-penetrating cargos for peptide-based PLK1 ligand delivery [34,35]. Thus, these multiple arginine serve a dual function as N-degrons and CPPs. This approach reinforces the potential of multiple arginines as key elements in PROTAC construction.

Synthesis of PROTAC and other peptides was performed using standard Rink amide resin-mediated solid-phase peptide synthesis (SPPS). During peptide sequence extension on resin support, EDCI and 1-hydroxy benzotriazole (HOBt) were used to couple amino acids in a DMF solvent. At each step, the Fmoc group was deprotected using 20% piperidine in DMF. Upon sequence completion, the peptides were cleaved from the resin using a cleavage mixture composed of trifluoroacetic acid, water, and triisopropylsilane (90:5:5, *v*/*v*/*v*, 2 mL) and subsequently precipitated in cold diethyl ether. Obtained crude peptides were purified using semi-preparative reverse-phase high-performance liquid chromatography (RP-HPLC), and the purity of synthesized peptides was verified via analytical HPLC. The molecular weights of synthesized peptides were determined using MALDI-TOF MS.

### 3.2. Validation of Arginine Recognition by UBR1

To determine the number of arginine residues required for UBR box recognition, a SAR study was performed. A series of peptides containing consecutive multiple arginine residues were synthesized, with an N-terminal lysine-anchored biotin attached at the C-terminus for UBR box detection (Figure 2b). Both UBR1 and UBR2 did not recognize peptides containing up to four arginine residues. Although UBR2 recognized peptides with R8 to R10, UBR1 Western blot intensities were not significant. Notably, peptides with Arg12 were well recognized by both UBR1 and UBR2. These results confirmed that Arg12 serves as a valid N-degron, making it a suitable component for PROTAC construction [19].

### 3.3. Recognition of UBR Box by NC1

**Figure 3:**
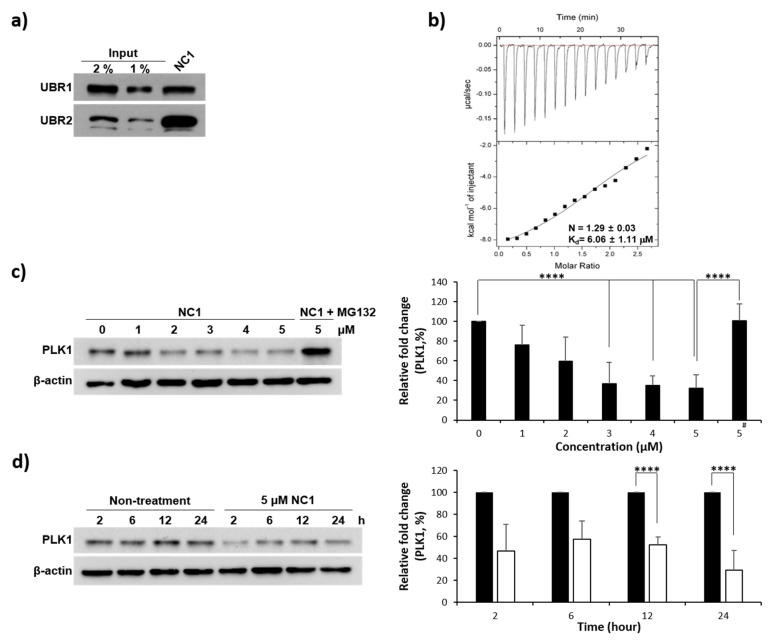
(**a**) Pull-down assay of UBR 1 and UBR2 via NC1. UBR1 and UBR2 were detected via immunoblotting. (**b**) Isothermal titration calorimetry (ITC) of the PLK1 PBD interaction with NC1. Raw ITC data (top panel) and integrated heats of injection (bottom panel) are presented for the titration between PLK1 PBD and NC1. In the bottom panel, the experimental data are shown as solid squares, and the least-squares best-fit curves derived from a simple one-site binding model are shown as a black line. (**c**) The effect of NC1 on PLK1 protein degradation in HeLa cells. Cells were treated with 0, 1, 2, 3, 4, and 5 µM of NC1 for 24 h, followed by treatment with 5 µM of MG132 for 8 h. PLK1 protein was assessed by immunoblotting. PLK1 degradation by NC1 was recovered by MG132 as a proteasome inhibitor. (**d**) The effect of NC1 on PLK1 protein degradation. **** *p* < 0.0005.

Following initial SAR validation of N-degrons, Arg12 was identified as a potential degron and incorporated at the N-terminus in PROTAC design. The PLH(n-octylphenyl)SpT (4j) was placed at the C-terminus and both warheads were connected via the Ahx linker. After constructing PROTAC NC1, we investigated its recognition by the UBR box. As hypothesized, NC1 was successfully recognized by both UBR1 and UBR2 (Figure 3a).

### 3.4. Evaluation of PLK1 PBD Binding Affinity

Prior to PLK1 degradation by NC1, the binding affinity between NC1 and PLK1 PBD was investigated using ITC, revealing a Kd of 6.06 µM (Figure 3b). In general, PROTACs function catalytically and recycle during degradation. Thus, transient PROTAC binding can still facilitate PROTAC-mediated protein degradation. This feature highlights a key advantage of PROTACs over traditional receptor-based inhibitors.

### 3.5. PLK1 Degradation

The PROTAC, NC1 was further evaluated for its ability to degrade the PLK1 protein. As shown in Figure 3c, cellular concentrations were measured via immunoblotting. Incubating NC1 with HeLa cells for 24 h demonstrated a dose-dependent PLK1 degradation, with the maximum degradation observed at 5 µM. To confirm that protein degradation occurred via the proteolysis pathway, the proteasome inhibitor MG132 was co-treated and the degradation process monitored. After 8 h, MG132 significantly reduced degradation, resulting in PLK1 retrieval (Figure 3c). This confirmed that PLK1 degradation was mediated by NC1 via the proteolysis pathway and not by PLK1 PBD warhead inhibition. PLK1 degradation began 2 h after NC1 treatment and persisted for 24 h (Figure 3d).

### 3.6. Complex Crystal Structure of PROTAC with PLK1 PBD

**Figure 4:**
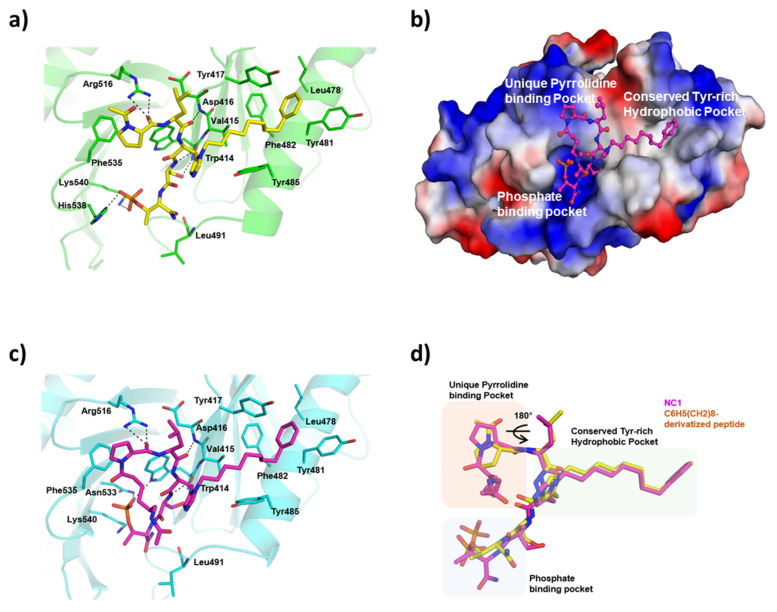
Crystal structure of the NC1 peptide bound to PLK1 PBD. (**a**) Crystal structure of PLH(n-octylphenyl)SpT (4j) in PBD of PLK1 (PDB ID 3RQ7) [11]. (**b**) Electrostatic potential surface of the PLK1 PBD crystal lattice with NC1 represented as a stick model in magenta; (**c**) NC1 interaction with amino acids present in the crystal lattice; (**d**) Comparison of binding parameters between PLH(n-octylphenyl)SpT (4j) and NC1 by viewing the overlapped crystal structure. Although pThr and C_6_H_5_(CH_2_)_8_ moieties are well defined in the electron density maps, the Arg12-linker is disordered. Only a portion of the linker is visible on the map, as it lacks a clear surface to bind to.

To further confirm PLK1 degradation resulted from selective binding of PLK1 PBD and UBR ligands in NC1 via ternary complex formation, the crystal PROTAC structure complexed with PLK1 PBD was investigated. As shown in Figure 4, the PLK1 PBD ligand in the PROTAC interacted similar to the potent pentapeptide, PLH(n-octylphenyl)SpT (4j) within the PBD crystal lattice. For example, the pyrrolidine in the PLK1 warhead of the PROTAC interacted with Phe535, Trp414, and Arg518 within the pyrrolidine binding pocket. Additionally, the long alkylphenyl chain at the histidine residue penetrated via a deep, conserved hydrophobic channel formed by tyrosine residues. The phosphate group of pT engaged in polar interactions with His538 and Lys540 within the phosphate binding pocket. These interactions are characteristic of PLH(n-octylphenyl)SpT (4j).

Ligands in the PLK1 PBD crystal lattice. Furthermore, an overlay view (Figure 4d) of the newly synthesized NC1 with PLH(n-octylphenyl)SpT (4j) confirmed that the ligands project toward the binding pockets and interact with residues in a similar pattern (Table S1). This finding showed that the PLK1 ligand in the PROTAC is capable of reaching its target and maintaining activity, even when integrated with a linker and an E3 ligase ligand.

### 3.7. Cell Viability Assay and Cancer Cell Morphology Analysis

**Figure 5:**
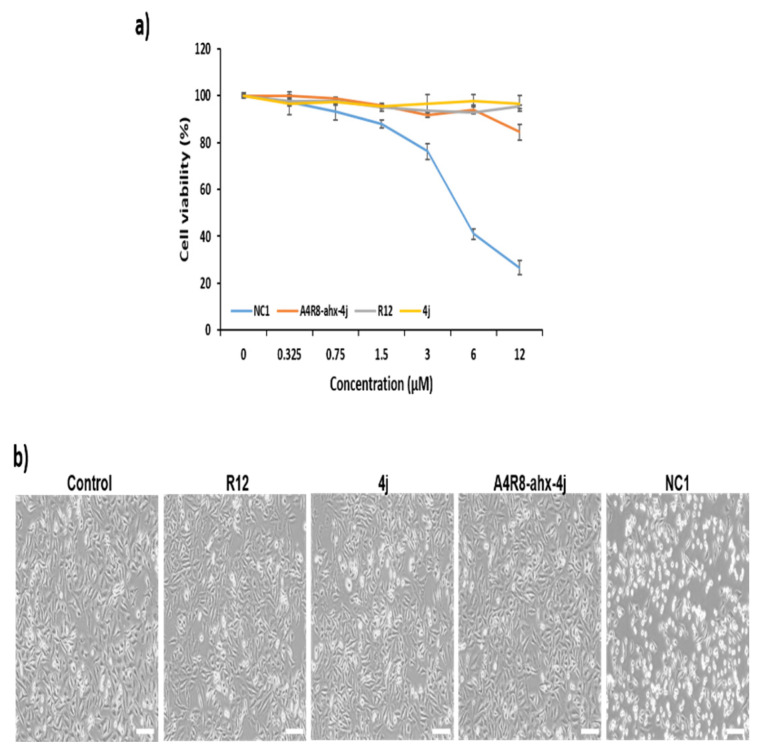
Changes in cell viability and morphological effects. (**a**) Cell viability effects of Arg12, PLH(n-octylphenyl)SpT (4j), A4R8-ahx-4j, and NC1 on HeLa cells. The effect of Arg12, PLH(n-octylphenyl)SpT (4j), A4R8-ahx-4j, and NC1 on HeLa cell viability was assessed using the MTT assay. HeLa cells were treated with increasing concentrations (0.325, 0.75, 1.5, 3, 6, and 12 µM) of each compound for 24 h. (**b**) Changes in HeLa cell morphology using Arg12, PLH(n-octylphenyl)SpT (4j), A4R8-ahx-4j, and NC1. Cells were incubated with 6 µM of Arg12, PLH(n-octylphenyl)SpT (4j), A4R8-ahx-4j, and NC1 for 30 h. Morphological changes were photographed using the phase-contrast microscope (×200 magnification, scale bar: 100 µm).

After successfully identifying NC1 as a PLK1 degrader, its inhibitory effects on cell proliferation were investigated. To assess this, an in vitro MTT assay on HeLa cells was conducted (Figure 5a). NC1treatment significantly decreased cell viability in a dose-dependent manner over 24 h. At 6 µM, NC1 reduced cell viability by >60%, and at 12 µM, a maximum reduction of 70% was observed. In contrast, Arg12, PLH(n-octylphenyl)SpT (4j), and the negative control A4R8-ahx-4j did not significantly affect cell viability, even at higher concentrations. The low inhibitory effect of the potent PLK1 PBD pentapeptide, PLH(n-octylphenyl)SpT (4j), can be attributed to its poor cell penetration owing to its peptide nature. Notably, NC1 exhibited an IC_50_ of 5.23 µM for cancer cell viability. This result showed the Arg12 N-terminal extension with the linker Ahx promoted the cell-penetrating ability of NC1. Thus, NC1 effectively inhibited cancer cell proliferation by penetrating Hella cell membranes.

Furthermore, changes in HeLa cell morphology were examined after NC1 treatment. HeLa cells were treated with Arg12, PLH(n-octylphenyl)SpT (4j), A4R8-ahx-4j, and NC1 and incubated for 30 h. Microscopic images revealed that most NC1 treated cells underwent significant morphological changes, becoming spherical (Figure 5b). In contrast, Arg12, PLH(n-octylphenyl)SpT (4j), and A4R8-ahx-4j did not induce notable morphological changes compared with the control. These findings indicate that NC1 has significant potential to penetrate cancer cells and induce morphological alterations.

### 3.8. Cell Penetrating Ability of FITC-Conjugated NC1 in Cancer Cells

**Figure 6:**
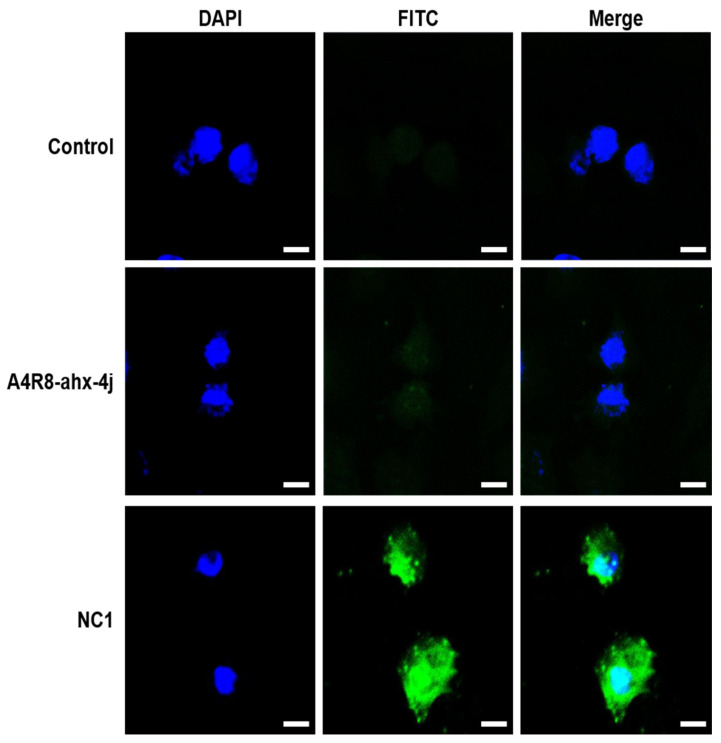
Confocal fluorescence microscopic images of FITC-conjugated A4R8-ahx-4j and FITC-conjugated NC1 in HeLa cells. Cells were incubated with 6 µM of FITC-conjugated A4R8-ahx-4j and FITC-conjugated NC1 for 24 h. The blue color indicates cell nuclei and the green color indicates FITC-conjugated compounds. The magnification of images is ×200 (scale bar: 25 µm).

The ability of the NC1 to penetrate the cell membrane was examined via fluorescence imaging analysis using FITC-conjugated NC1. As shown in Figure 6, confocal microscopy revealed green fluorescence from FITC-conjugated NC1 and blue fluorescence from 4′,6-diamidino-2-phenylindole (DAPI)-stained chromosomes. The merged images demonstrated the colocalization of FITC-conjugated NC1 with chromosomes, indicating that NC1 effectively penetrated the cytosol and reached the nucleus. In contrast, FITC-conjugated A4R8-ahx-4j did not exhibit significant cell membrane penetration. These findings indicate that despite being a peptide, NC1 can penetrate the cell membrane and reach the nucleus, owing to the cell-penetrating ability of Arg12 and ahx linker. Thus, NC1 has the potential to evolve as a therapeutic agent for cancer treatment.

### 3.9. G2/M Phase Cell Cycle Arrest Using a Fluorescence-Activated Cell Sorter

**Figure 7:**
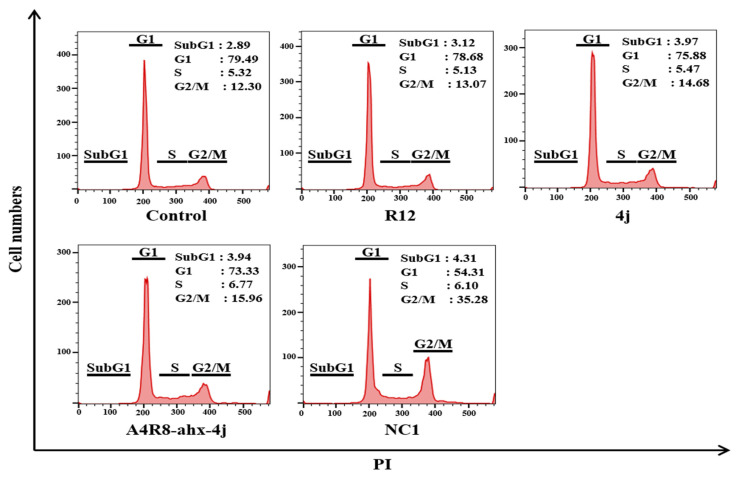
Detection of cell cycle arrest in HeLa cells treated with 6 µM of Arg12, PLH(n-octylphenyl)SpT, A4R8-ahx-4j, and NC1 for 24 h. Cells were stained with propidium iodide (PI) and analyzed using flow cytometry.

To investigate the effect of NC1 on cancer cell proliferation, cell cycle distribution analysis was performed using flow cytometry. Cells were individually treated with Arg12, PLH(n-octylphenyl)SpT, A4R8-ahx-4j, and NC1 at 6 µM for 24 h. As depicted in Figure 7, Arg12 (0.77%), PLH(n-octylphenyl)SpT (2.38%) and the negative control A4R8-ahx-4j (3.66%) caused no notable changes in the G2/M phase of cell cycle arrest compared with the control. However, NC1 induced a 22.98% marked increase in the G2/M cell population. This partial yet significant increase by NC1 was sufficient to induce cell cycle arrest at the G2/M phase.

### 3.10. Apoptosis Effects of NC1 on Cancer Cells

**Figure 8:**
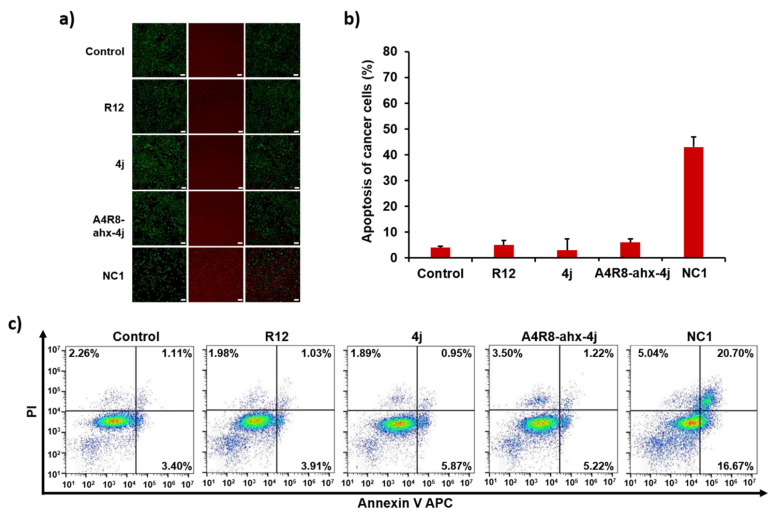
(**a**) Fluorescence images show cell apoptosis after treatment with 6 µM of Arg12, PLH(n-octylphenyl)SpT (4j), A4R8-ahx-4j, and NC1 for 24 h. The green color represents live cells and the red color represents dead cells (×40 magnification, scale bar: 100 µm). (**b**) Quantification of apoptotic cells. (**c**) FACS analysis expresses apoptosis quantification in HeLa cells after treatment with 6 µM of Arg12, PLH(n-octylphenyl)SpT (4j), A4R8-ahx-4j, and NC1 for 24 h. All diagrams show the apoptotic cell population via double staining with Annexin V APC and PI.

Owing to the significant potential of NC1 to induce cell cycle arrest, we considered investigating its effects on apoptosis in cancer cells using a fluorescence apoptosis assay and FACS analysis. In the fluorescence apoptosis assay, live cells were stained green and dead cells were stained red (Figure 8). Arg12, PLH(n-octylphenyl)SpT (4j), and A4R8-ahx-4j exhibited a pattern similar to the control, showing few apoptotic red cells in the fluorescence images.

In contrast, NC1 displayed a noticeably higher number of dead cells owing to apoptosis, approximately 10 times more than the control (Figure 8b). To quantify apoptosis, FACS analysis with double staining using Annexin V APC and PI was performed (Figure 8c). FACS analysis revealed that NC1 significantly increased early-phase apoptosis by 13.27% and late-phase apoptosis by 19.59% compared with the control (Figure 8c). Collectively, these results indicate that NC1 promotes PLK1 degradation, subsequently inducing cell cycle arrest and apoptosis.

### 3.11. Anti-Tumorigenic Effect of NC1 on Tumor-Bearing Mice

**Figure 9:**
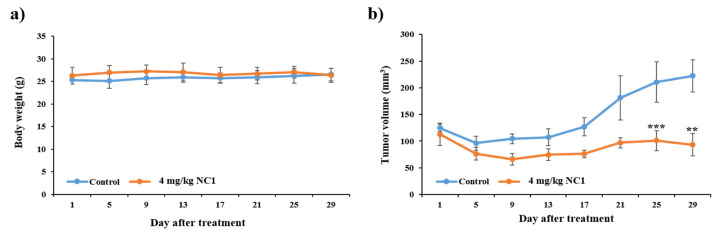
In vivo anticancer effect of NC1 on heterotopic HeLa xenograft cervical cancer. In total, 5 × 10^6^ cells were subcutaneously inoculated into the right flank. (**a**) Average body weight changes were monitored in the control and NC1-treated groups over 29 d. (**b**) Tumor volume changes were observed after NC1 treatment (4 mg/kg body weight) for 29 d, with measurements recorded at 3–4 d intervals. ** *p* < 0.005 *** *p* < 0.001. Values represent the average ± SEM (n = 4 per group). Tumor volumes were calculated using the formula (length × width^2^ × 0.52), and growth curves were plotted accordingly.

Owing the in vitro effects of NC1, we evaluated its anti-tumor activity using an in vivo tumor mouse model. Mice bearing HeLa xenograft cervical cancer were injected via tail vein with PBS or NC1 for 29 d (n = 4), as shown in Figure 9b. This figure shows a substantial increase in tumor growth in the PBS-treated group. In contrast, the NC1-treated group exhibited a significant reduction in tumor size. This effect can be attributed to effective PLK1 degradation via the N-degron pathway. To determine if the change in tumor size was due to systemic shock, we periodically monitored body weight of tumor-bearing mice before administering NC1. As shown in Figure 9a, the body weight of mice remained constant throughout the experiment, indicating that tumor size reduction was solely due to the anti-tumorigenic activity of NC1.

## 4. Discussion and Conclusions

Cervical cancer remains a major global health issue, with most deaths occurring in low- and middle-income countries. Chemoresistance to standard treatments, such as cisplatin, emphasizes the need for new therapies [36]. PLK1, a key mitotic regulator, is often overexpressed in cervical cancer, making it a promising target [37]. Although kinase inhibitors are effective, their use is limited by off-target toxicity [38]. PLK1 PBD-based PROTACs offer a more selective approach by targeting the allosteric PBD site. PROTAC technology facilitates targeted protein degradation via the ubiquitin-proteasome system (UPS), overcoming resistance and minimizing toxicity. Acting catalytically at low concentrations, PROTACs enhance selectivity and reduce treatment costs [39]. This approach is particularly effective for modulating difficult protein–protein interactions (PPIs) that are often challenging to target with small molecules.

We developed peptide-based PROTACs that function via the N-degron pathway. Our design incorporates multiple successive Arg12 residues, serving as an Arg/N-degron targeting UBR protein, a type of E3 ligase common in most human cells and organs. This approach addresses the major limitation of PROTACs—limited scope and poor E3 ligase expression in certain organs. In addition, Arg12 functions as an intrinsic cell-permeable cargo, eliminating the need for external carriers, such as CPPs, polyethylene glycol (PEG), or lipid nanoparticles. Arg12′s cell-penetrating ability in this PROTAC was confirmed using FITC-conjugated NC1 penetrating the cytosol and nucleus of HeLa cells.

To date, although PROTACs have been explored for immune disorders, viral infections, and neurodegenerative diseases [15], cancer remains the most studied area. Cancer-related PROTAC research primarily targets BET proteins, transmembrane receptor tyrosine kinases (RTKs), and kinase families [13]. Among these kinases, PLK1 remains the least explored, with only a few reports, including one from our group [40]. Another study used the kinase domain-targeting ligand BI2536 to target acute myeloid leukemia. The primary disadvantage of kinase domain-targeting inhibitors is their lack of selectivity, owing to similar ATP-binding sites among related kinases, such as PLK2 and PLK3 [41]. In contrast, our study employed the potent and PLK1-selective PBD inhibitor PLH(n-octylphenyl)SpT to construct NC1, addressing selectivity issues. Thus, we derived the crystal structure of NC1 (POI warhead) bound to PLK1 PBD, replicating the key binding parameters of PLH(n-octylphenyl)SpT [11] with PBD. NC1 facilitated dose-dependent PLK1 degradation, which was reversed by the proteasome inhibitor MG132. These findings indicate that NC1 degraded PLK1 via the ubiquitin-mediated protein degradation pathway. Consequently, NC1 inhibited in vitro HeLa cell proliferation by inducing G2/M cell cycle arrest and subsequent apoptosis via PLK1 degradation. In vivo studies confirmed the therapeutic potential of NCI by reducing tumor size and weight, validating NC1 as an effective tool for degrading PLK1 in cancer cells.

In conclusion, our study identified NC1, a peptide-based PROTAC that depletes PLK1 in cancer cells via the Arg/N-degron pathway. Key features include the use of Arg/N-degron Arg12, which functions both as a degron and a CPP; the design of a peptide-based PROTAC targeting PLK1 via PBD binding, utilizing UBR box as the E3 ligase for degradation; and the potent in vitro anticancer activity observed by inhibiting cell proliferation, inducing G2/M cell cycle arrest, and triggering subsequent apoptosis. Furthermore, NC1 notably reduced tumor size in HeLa mouse xenograft models at micromolar concentrations. These findings support NC1 as a promising therapeutic candidate for cervical cancer treatment via PLK1 degradation. This study highlights the potential of N-degron pathway-based PROTAC technology as an effective alternative to traditional chemotherapy in cervical cancer drug discovery.

## Data Availability

The data supporting the findings of this study are available from the corresponding author upon request.

## Supplementary Materials

The following supporting information can be downloaded at: https://www.mdpi.com/article/10.3390/pharmaceutics17081027/s1.
