# Supplementary material for: N-Degron-Based PROTAC Targeting PLK1: A Potential Therapeutic Strategy for Cervical Cancer
Source: Pharmaceutics. 2025 Aug 7;17(8):1027. doi: 10.3390/pharmaceutics17081027 (PMC12389170; doi:10.3390/pharmaceutics17081027)
Supplement: Supplementary file 1 [file pharmaceutics-17-01027-s001.zip › pharmaceutics-3772557-supplementary.pdf]

Supplementary Materials

N-degron-based PROTAC Targeting PLK1: A Potential Therapeutic Strategy for Cervical Cancer

Pethaiah Gunasekaran [1,2,†], Sang Chul Shin [3,†], Yeon Sil Hwang [2], Jihyeon Lee [2,4], Yeo Kyung La [2], Min Su Yim [5], Hak Nam Kim [1], Tae Wan Kim [6], Eunjung Yang [7], Soo Jae Lee [4], Jung Min Yoon [8], Eunice EunKyeong Kim [8], Seob Jeon [7], Eun Kyoung Ryu [1,*] and Jeong Kyu Bang [1,2,*]

1 Division of Magnetic Resonance, Korea Basic Science Institute (KBSI), Ochang, Cheongju 28119, Republic of Korea; gunaharaks@gmail.com (P.G.)

2 Dandicure Inc., Ochang, Cheongju 28119, Republic of Korea; yshwang2@dandicure.com (Y.S.H.); jhlee4@dandicure.com (J.L.)

3 Convergent Research Support Division, Technological Convergence Center, Korea Institute of Science and Technology (KIST), Seoul 02792, Republic of Korea.; scshin84@kist.re.kr

4 College of Pharmacy, Chungbuk National University, Cheongju 28160, Republic of Korea

5 Korea Disease Control and Prevention Agency, National Institute of Health Center for Emerging Virus Research, Division of Emerging Virus and Vector Research, Cheongju, 28159,Republic of Korea

6 Future Innovation Medical Research Center, Soonchunhyang University Cheonan Hospital, Cheonan 31151, Republic of Korea

7 Department of Obstetrics and Gynecology, College of Medicine, Soonchunhyang

University Cheonan Hospital, Cheonan 31151, Republic of Korea; sjeon4595@gmail.com (S.J.)

8 Biomedical Research Institute, Korea Institute of Science and Technology, Seoul 02792, Republic of Korea; eunice@kist.re.kr (E.E.K.)

* Correspondence: ekryu@kbsi.re.kr (E.K.R.); bangjk@kbsi.re.kr (J.K.B.); Tel.: +82-43-240-5023; Fax: +82-43-240-5059

†These authors contributed equally to this work.

Figure S1. Uncropped full-length picture of western blotting membrane. Uncropped full-length pictures of western blotting membranes in Fig.2b presented in the supplementary Fig. 1.

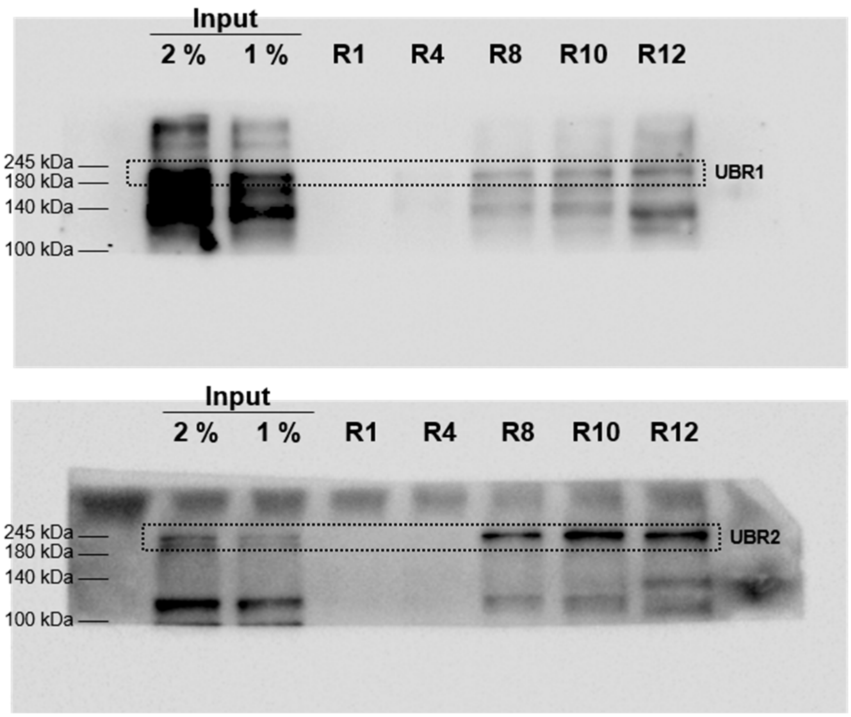

Figure S2. Uncropped full-length picture of western blotting membrane. Uncropped full-length pictures of western blotting membranes in Fig.3a presented in the supplementary Fig. 2.

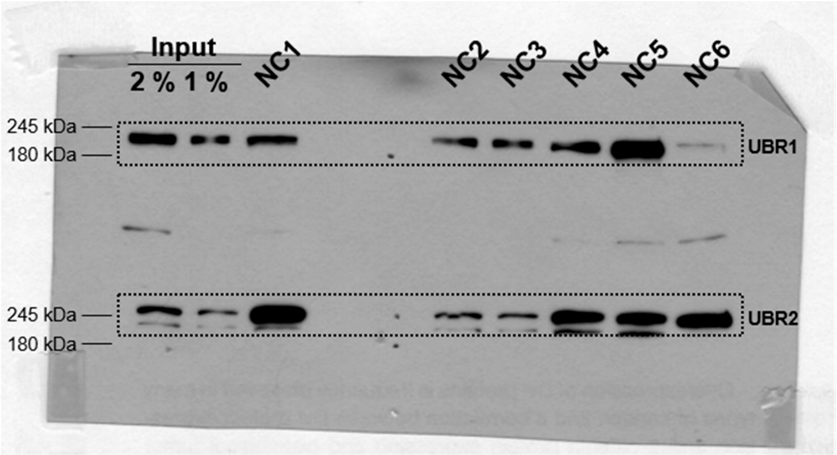

Figure S3. Uncropped full-length picture of western blotting membrane. Uncropped full-length pictures of western blotting membranes from repeat experiments in Fig.3c presented in the supplementary Fig. 3.

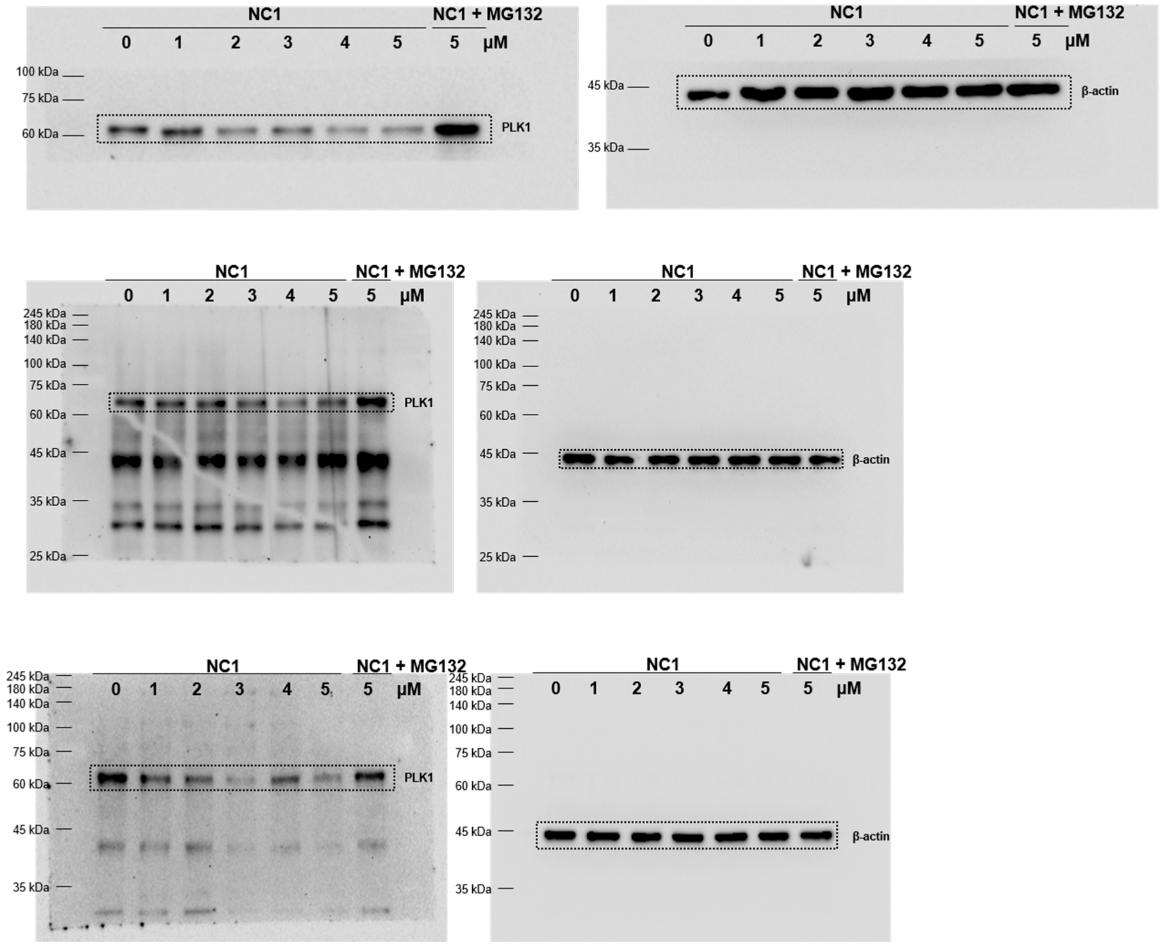

Figure S4. Uncropped full-length picture of western blotting membrane. Uncropped full-length pictures of western blotting membranes from repeat experiments in Fig.3d presented in the supplementary Fig. 4.

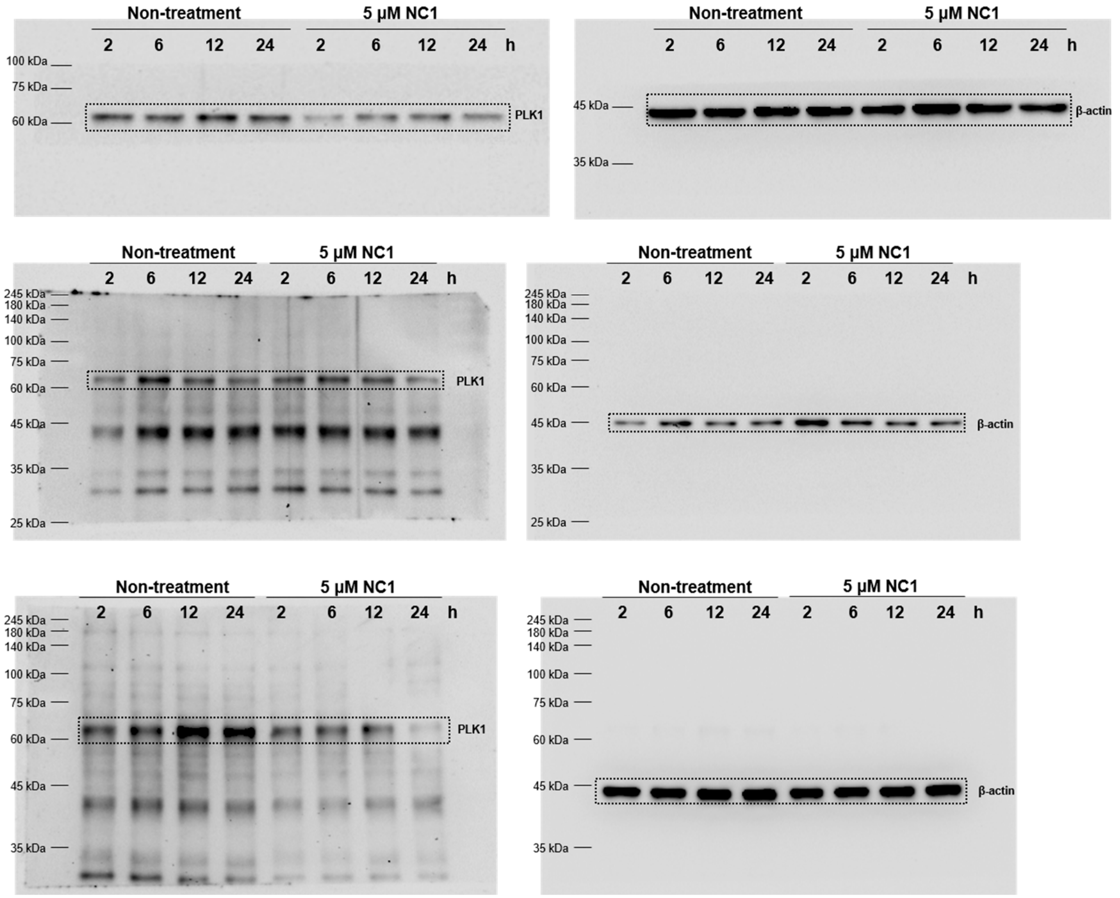

Figure S5. Raw low-concentration ITC data. Raw ITC data used for model fitting in Fig. 2b. Each value represents heat change per injection and related fitting parameters.

	DH	INJV	Xt	Mt	XMt	NDH	Fit	DY
1	-3.97804	2.5	0	0.01	0.12306	-15912.2	-15969.4	57.25122
2	-3.95386	2.5	0.00122	0.00988	0.24761	-15815.4	-15520.3	-295.096
3	-3.81177	2.5	0.00242	0.00976	0.37366	-15247.1	-15005.5	-241.616
4	-3.62975	2.5	0.0036	0.00964	0.5012	-14519	-14419.1	-99.8773
5	-3.37268	2.5	0.00477	0.00952	0.63025	-13490.7	-13757.5	266.8314
6	-3.18878	2.5	0.00593	0.00941	0.76078	-12755.1	-13020.2	265.0569
7	-2.9403	2.5	0.00707	0.00929	0.89282	-11761.2	-12211	449.8279
8	-2.74534	2.5	0.0082	0.00918	1.02634	-10981.3	-11339.9	358.5571
9	-2.62798	2.5	0.00931	0.00907	1.16137	-10511.9	-10422.8	-89.1078
10	-2.38911	2.5	0.0104	0.00896	1.29789	-9556.46	-9481.17	-75.2885
11	-2.28158	2.5	0.01148	0.00885	1.43591	-9126.31	-8539.62	-586.691
12	-2.1132	2.5	0.01255	0.00874	1.57542	-8452.81	-7623.09	-829.713
13	-1.71013	2.5	0.0136	0.00863	1.71643	-6840.54	-6753.57	-86.9649
14	-1.42962	2.5	0.01464	0.00853	1.85893	-5718.49	-5947.74	229.2516
15	-1.10041	2.5	0.01566	0.00842	2.00293	-4401.66	-5215.89	814.2302
16			0.01666	0.00832				

Table S1 Statistics on data collection and refinement

	Human_PLK1PBD with NC1 compound
**Data collection statistics**	
X-ray source	PLS_5C
Wavelength, Å	0.97942
Space group	$P2_1$
Unit cell parameters	
a, b, c (Å)	35.67; 51.45; 57.41
α, β, γ (°)	90.0; 100.89; 90.0
[a]Resolution, Å	50.0-1.95 (2.02–1.95) [a]
No. of total reflections	275366
No. of unique reflections	14849
[a]Completeness, %	97.6 (95.0)
[a]$I/\sigma(I)$	24.6 (2.6)
[a]Redundancy	4.9 (3.4)
[b]R_{merge}, %	9.8 (36.6)
[a]$CC_{1/2}$, %	98.7 (66.7)
**Refinement**	
Resolution, Å	38.0–1.95 (2.02–1.95) [a]
[c]R_{cryst}/R_{free}, %	17.8/23.6
No. of protein atoms	1767
No. of water molecules	69
No. of ligand molecules	1
Bond lengths, Å	0.008

Bond angles, °	1.330
Average *B*-factor, Å^2	29.5
Ramachandran analysis	
Favored, %	95.79
Allowed, %	4.21
Outliers	0
Rotamer outliers, %	0.51
PDB entry	9IM7

[a] Values in parentheses represent the outer most resolution shell.

[b] $R_{merge} = \sum_h \sum_i |I(h,i) - \langle I(h) \rangle| / \sum_h \sum_i I(h,i)$, where $I(h,i)$ is the intensity of the ith measurement of reflection h and $\langle I(h) \rangle$ is the mean value of $I(h,i)$ for all i measurements.

[c] R_{free} was calculated from a randomly selected 5% set of reflections not included in the R value calculation.
